# Integrated omics approaches for flax improvement under abiotic and biotic stress: Current status and future prospects

**DOI:** 10.3389/fpls.2022.931275

**Published:** 2022-07-25

**Authors:** Bindu Yadav, Vikender Kaur, Om Prakash Narayan, Shashank Kumar Yadav, Ashok Kumar, Dhammaprakash Pandhari Wankhede

**Affiliations:** ^1^Division of Germplasm Evaluation, ICAR-National Bureau of Plant Genetic Resources, New Delhi, India; ^2^College of Arts and Sciences, University of Florida, Gainesville, FL, United States; ^3^Division of Genomic Resources, ICAR-National Bureau of Plant Genetic Resources, New Delhi, India

**Keywords:** abiotic and biotic stress, climate change, flax, fungal diseases, omics, linseed

## Abstract

Flax (*Linum usitatissimum* L.) or linseed is one of the important industrial crops grown all over the world for seed oil and fiber. Besides oil and fiber, flax offers a wide range of nutritional and therapeutic applications as a feed and food source owing to high amount of *α*-linolenic acid (omega-3 fatty acid), lignans, protein, minerals, and vitamins. Periodic losses caused by unpredictable environmental stresses such as drought, heat, salinity-alkalinity, and diseases pose a threat to meet the rising market demand. Furthermore, these abiotic and biotic stressors have a negative impact on biological diversity and quality of oil/fiber. Therefore, understanding the interaction of genetic and environmental factors in stress tolerance mechanism and identification of underlying genes for economically important traits is critical for flax improvement and sustainability. In recent technological era, numerous omics techniques such as genomics, transcriptomics, metabolomics, proteomics, phenomics, and ionomics have evolved. The advancements in sequencing technologies accelerated development of genomic resources which facilitated finer genetic mapping, quantitative trait loci (QTL) mapping, genome-wide association studies (GWAS), and genomic selection in major cereal and oilseed crops including flax. Extensive studies in the area of genomics and transcriptomics have been conducted post flax genome sequencing. Interestingly, research has been focused more for abiotic stresses tolerance compared to disease resistance in flax through transcriptomics, while the other areas of omics such as metabolomics, proteomics, ionomics, and phenomics are in the initial stages in flax and several key questions remain unanswered. Little has been explored in the integration of omic-scale data to explain complex genetic, physiological and biochemical basis of stress tolerance in flax. In this review, the current status of various omics approaches for elucidation of molecular pathways underlying abiotic and biotic stress tolerance in flax have been presented and the importance of integrated omics technologies in future research and breeding have been emphasized to ensure sustainable yield in challenging environments.

## Introduction

**Figure 1:**
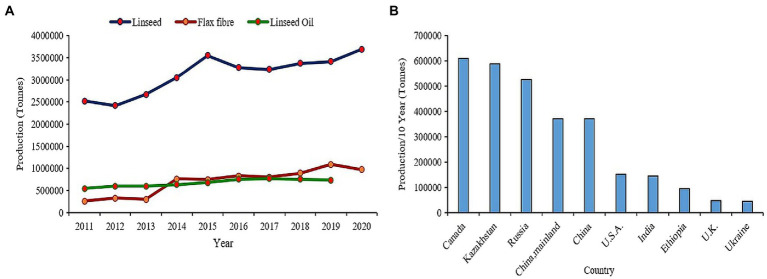
**(A)** Trends in global production of linseed and flax fiber in past decade. **(B)** Flaxseed production in top 10 countries in the world.

Flax (*Linum usitatissimum* L.) or linseed is one of the primeval crops domesticated for oil and fiber since beginning of civilization (Zohary and Hopf, 2000). It is believed to be originated in either the Middle East or Indian regions from where it spread to whole world (Vavilov, 1951; Green et al., 2008). Since ages, the oil from flax seed has been used in paints, varnishes, and polymer industries owing to its unique fatty acid composition (Przybylski, 2005; Shim et al., 2015) while the fiber extracted from flax stem has been used in textile industry to produce quality Linen fabrics. Nutritionally flaxseeds are very dense as they are packed with high amount of alpha linolenic acid (55–57%), proteins (upto 18.29%), fibers (27.3%), vitamin B1, and lignans particularly secoisolariciresinol diglucoside (SDG; 294–700 mg/100 g) making it among preeminent functional food (Singh et al., 2011; Goyal et al., 2014; Kajla et al., 2015). Flax seed consumption has proven beneficial effects on coronary heart disease, cancer, neurological/hormonal disorders, and atherosclerosis (Westcott and Muir, 2003; Hosseinian et al., 2006; Bassett et al., 2009). Presently, China occupies the paramount position in terms of flax consumption and is the largest importer valuing 31,108 M US$ in the past decade which accounts for 26.8% of total global flax import in the year 2020. Canada is the leading producer and exporter of flax worldwide over the past decade, while India ranks seventh in terms of production and eleventh in terms of export (FAOSTAT, 2022; Figure 1). Biotic and abiotic stress factors have been the major constraints in increasing flax production worldwide. The productivity of fiber flax is severely affected by devastating fungal diseases such as *Fusarium* wilt, *Alternaria* blight, powdery mildew, rust, and pasmo in European countries, whereas the oil type linseed mainly cultivated in Asian countries, particularly India suffers from drought, salinity, and heat in conjugation to varied diseases and insect-pests. In addition, the warmer climate of these tropical countries is not suitable for fiber flax which requires a prolonged cool season for effective yields and fiber quality. As a result, yields have been stagnated in these countries. The renewed interest in flax consumption as functional food has led to the increase in consumer demand for flax-based products such as multigrain breads, ready-to-eat breakfast cereals, breakfast drinks, salad dressings, biscuits, crackers, soups, and cakes (Coşkuner and Karababa, 2007; Ayelign and Alemu, 2016). Moreover, with the recent advances in material science, the flax fiber has new range of industrial applications, such as geotextiles, biopolymers, specialty papers, composites, and biofuels (Diederichsen and Ulrich, 2009; Cullis, 2011), and has gained new attention because of its quality, biodegradability, and recyclability. Thus, the burgeoning interest revolving around health promoting effects and natural fiber industry has fueled for enhanced demand worldwide. The increased demand is reflected by the up-scaling trend in global production of linseed from 2.5 million tonnes to more than 3.5 million tonnes as well as flax fiber from about 26,000 tonnes to approx. 1 million tonnes over past decade (FAOSTAT, 2022; Figure 1). However, environmental challenges, such as dwindling water resources, salinization or alkalinization of soil, extreme temperature fluctuations, fungal diseases, such as wilt, rust, and pasmo, have deleterious effects on plant growth resulting in huge yield loss in flax (Fofana et al., 2006; Saha et al., 2021; Zare et al., 2021).

Among abiotic stresses, drought is one of the most prevalent and detrimental constraints to agricultural production, that negatively affects the overall crop growth, yield, and quality causing more than 50% average yield loss (Fahad et al., 2017; Kole, 2020). It is expected to wreak havoc on plant growth on more than half of arable land by 2050 (Jaggard et al., 2010). Western Canada, Russia, China, and India are important flax producing regions and during the last 100 years, annual precipitation has become less evenly distributed in these areas in addition to temperature change (Gitay et al., 2002). Scarcity of soil moisture can negatively impair the yield potential, oil content and fatty acid composition, and fiber quality traits in flax (Fofana et al., 2006; Abd El-Fatah, 2007; Heller and Byczyńska, 2015). Drought results in reduced leaf expansion, leaf senescence, abscission, oxidative damage, and increased membrane lipid peroxidation thereby disrupting normal metabolism (Hu and Xiong, 2014). Although flax tolerates drought better than many other oil and food crops due to its hardiness; however, at the same time, flax plants transpire very high amounts of water owing to high transpiration coefficient (the amount of water necessary to produce one unit of dry matter) value of 787–1,093 (Kozłowska, 2007; Heller and Byczyńska, 2015). Therefore, fiber flax requires annual precipitation of at least 600–650 mm for optimal yields, of which at least 110–150 mm of rain fall is essential in the vegetation period. Thus, water scarcity continues to be a significant impediment to flax production as it is a neglected crop in developing countries and is normally cultivated in rain-fed areas with poor management and low input conditions (Lisson and Mendham, 2000; Dash et al., 2014; Kaur et al., 2017). Drought is an erratic and highly unpredictable environmental phenomenon; therefore, selection should target drought tolerant genotypes having yield potential. Accordingly, long-term traditional breeding programs and later development of transgenic flax were initiated to combat these constraints and improve flax production (Tawfik et al., 2016). Since drought tolerance is a complex polygenic trait, understanding the adaptive mechanisms and identification of underlying genes/markers/QTLs could pave a way for genetic enhancement and productivity of flax in arid and semi-arid regions. Only a few studies have been reported identifying drought resilient genotypes in flax (Diederichsen et al., 2006; Qi et al., 2010; Sharma et al., 2012; Asgarinia et al., 2017) and genome-wide analysis of drought induced gene expression (Dash et al., 2014). The root system is shallow in flax compared to other oilseed crops such as rapeseed, sunflower, and safflower. Therefore, studying root system architecture is of pivotal importance for more efficient water acquisition in flax. The importance of root traits for efficient water and nutrient absorption under water scarce conditions have been realized recently in many crops, such as rice, wheat, and maize (Tuberosa et al., 2002; Manschadi et al., 2006; Gowda et al., 2011; Kaur et al., 2020); however, knowledge is still limited in flax (Soto-Cerda et al., 2019, 2020).

Soil salinity has risen exponentially in recent years due to a number of factors including excessive irrigation, low precipitation, high surface evaporation, rock weathering, ion exchange, and poor cultural practices (Bui, 2020; Dubey et al., 2020). Approximately 20% of total cultivated and 33% of irrigated land is currently affected by saline conditions, and more than 50% of arable land is predicted to be salinized by 2050 (Jamil et al., 2011; Shrivastava and Kumar, 2015). In flax, soil salinity-alkalinity leads to delayed germination and emergence, low seedling survival, irregular crop growth, and lower yield (Dubey et al., 2020). Few studies have reported screening of flax germplasm against salinity-alkalinity stresses (Kaya et al., 2012; Patil et al., 2015; Nasri et al., 2017; El-Afry et al., 2018; Kocak et al., 2022) and identified salinity tolerant lines based on germination, seedling characteristics, and biomass and K^+^/Na^+^ ratio. Genes conferring salt tolerance by increasing root length, improving membrane injury and ion distribution in flax were identified by Wu et al. (2019a). Since flax can tolerate the pH up to 9, thus can serve to utilize agricultural land where other crops cannot be successfully grown.

Heat stress adversely affects the growth, development, and physiological processes, and thus yield particularly in tropical and subtropical regions (Ramirez-Villegas et al., 2020). A sustained period of heat stress (40°C for 5–7 days) during flowering might have a significant impact on pollen production, pollen viability, flowering, boll development, seed set, oil quality, and quantity in flax (Cross, 2002; Cross et al., 2003; Saha et al., 2019, 2021). Fiber flax does not require high temperatures. The largest and highest quality fiber flax yields are obtained in humid, cloudy, and relatively cool (18°C–20°C) conditions. High temperature particularly terminal heat is limiting for flax growth, resulting in low adaptation of elite fiber flax genotypes to warmer climes. Although few studies have been conducted on the effects of higher temperatures on growth, physiological processes, and yields in flax, the molecular dissection is hitherto unknown (Cross et al., 2003; Pokhrel and Meyers, 2022).

Among biotic stresses, globally most widespread and devastating pathogen of flax is *Fusarium oxysporum* f. sp. *lini* which causes wilt disease and can result in an 80%–100% loss in yield (Rashid, 2003). The fungus infiltrates into the flax root cells and then advances intra-cellularly into vascular tissue. The fungal microconidia germinate and thus block the vascular vessels and prevent water and nutrient translocation resulting in epinasty followed by progressive wilting and death. Along with fusarium wilt, flax rust, caused by *Melampsora lini* is another important fungal disease limiting flax production worldwide. The gene-for-gene relationship was initially described for the flax rust interaction (Flor, 1956). Since then, it has served as a model pathosystem to study underlying genetics in host-pathogen interaction in plants. Extensive work has been done on flax-rust interaction at molecular (resistance gene *R*) and pathogen effectors (avirulence genes *Avr*) level (Ravensdale et al., 2011); however, whole genome responses involving signaling and defense remains largely unexplored. In addition to wilt and rust, other widespread disease of flax is pasmo caused by *Septoria linicola*, while anthracnose and powdery mildew (caused by *Colletotrichum lagenarium* and *Oidium lini*, respectively) are less common and endemic in nature.

Flax occupies an important position in global economy due to its wide industrial utility as well as regional and niche preferences. However, unprecedent climate changes may have detrimental impact on flax productivity, and therefore in depth understanding of various diseases and environmental stresses assumes importance for future planning from the perspective of growth, equity and sustainability. Recent technological advances in DNA sequencing and molecular biology have expedited genomics and transcriptomic research and thus paved way for accelerated development of other domains of omics such as proteomics, metabolomics, and phenomics. Amalgamation of omics assisted multidisciplinary approach is necessary for understanding and investigating complex stress tolerance mechanism to design climate resilient flax varieties. Despite multitudinous utility and being a model crop for research studies, there is scanty and scattered information regarding integration of omics approaches for flax improvement. Present review is intended to apprise the readers about the current status of omics interventions in flax in response to major biotic and abiotic stresses and underlying molecular pathways.

## Integrated omics approaches in technological era

**Figure 2:**
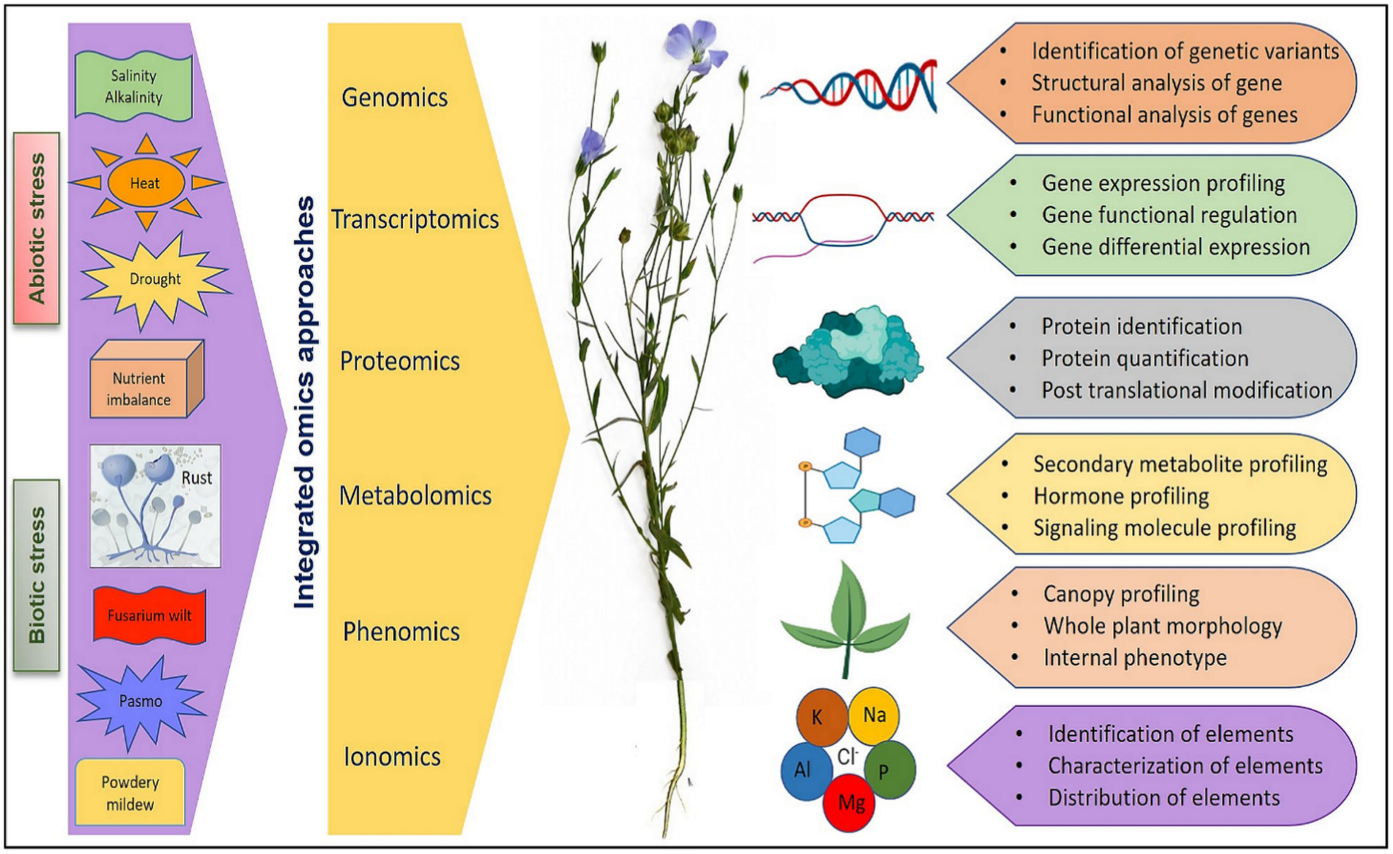
An overview of integration of different omics approaches for flax improvement under various abiotic and biotic stresses.

Major components of omics include genomics (generation of genetic and genomic resources, gene mapping, functional genomics, and genomic selection), transcriptomics (gene regulation and expression profiling), proteomics (protein identification and effects), metabolomics (metabolite profiling, regulation, pathway and intermediates), phenomics (automated study and analysis of phenotypic and physiological effects), and ionomics (elemental identification, composition, effects, and interactions). Different omics mechanism and their integration has pivotal role in understanding plant systems biology as elaborated in extensive reviews (Fukushima et al., 2009; Weckwerth et al., 2020; Pazhamala et al., 2021). Omics assisted technologies have been advocated and utilized for engineering stress tolerance in reviews on rice (Kumari et al., 2022), wheat (Shah et al., 2018), soybean (Chaudhary et al., 2015), tomato (Chaudhary et al., 2019), and flax (Shivaraj et al., 2019). However, relatively less efforts have been made to utilize the available genetic and genomic resources for flax improvement compared to other crops. The advanced tools like genome-wide association studies (GWAS) and genomic selection in conjugation with other omic technologies provide an opportunity to increase the precision of plant selection for flax improvement as suggested by Shivaraj et al. (2019) and Akhmetshina et al. (2020) while reviewing the utilization of high-throughput sequencing technologies and omics-assisted breeding for development of climate-smart flax. Therefore, a holistic approach involving diverse technologies can greatly facilitate the introduction of climate-resilient traits into flax genotypes for sustainable productivity. A schematic view of integration of key omics approaches that can be utilized for the improvement of flax under various biotic and abiotic stresses is presented in Figure 2. In further sections of the review, we have elaborated the advancement made in various omic technologies and the amalgamation of omics data in future flax breeding for economic and sustainable yield.

## Genomics

**Figure 3:**
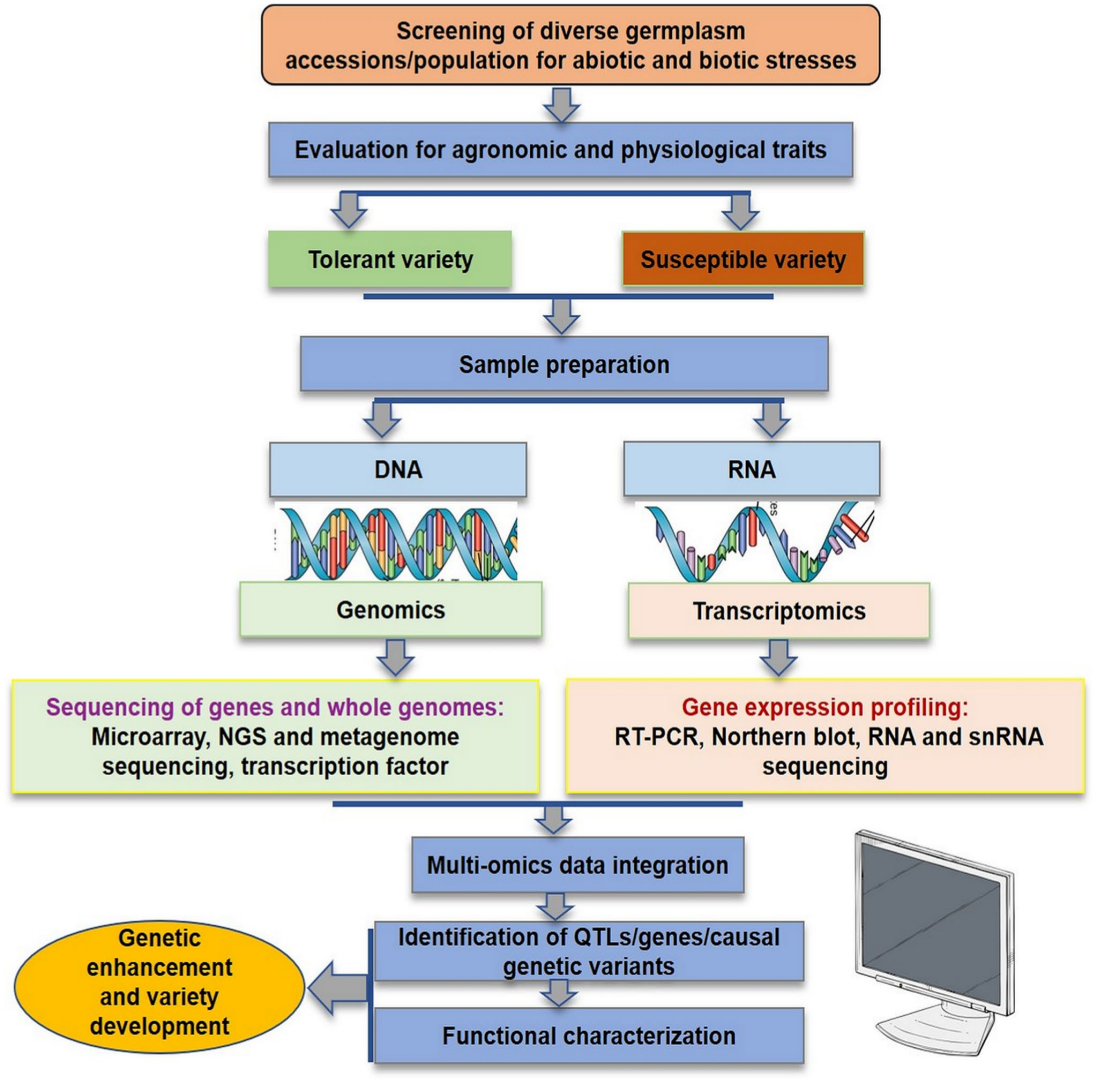
Genomics enabled strategies for flax improvement in response to adverse climatic conditions and pathogenic invasion.

In the initial years of the century, many molecular markers such as rapid amplification of polymorphic DNA (RAPD), amplified fragment length polymorphism (AFLP), inter-simple sequence repeat (ISSR), and expressed sequence tags-simple sequence repeats (EST-SSR) were used to assess the genetic diversity in flax (Oh et al., 2000; Fu et al., 2002a,b, 2003; Green et al., 2008; Cloutier et al., 2009, 2011, 2012; Rajwade et al., 2010; Uysal et al., 2010; Kaur et al., 2018; Saroha et al., 2022a). The substantial lead in the generation of genomic resources was made with the availability of whole genome sequence of flax (Wang et al., 2012). Subsequently, the whole genome resequencing and reduced representation sequencing information has been effectively utilized to understand crop diversity, marker identification, linkage map construction and QTL identification in flax. Genome wide SNP discovery through genotyping-by-sequencing (GBS) approach has been used to identify 258,873 SNPs distributed on all 15 flax chromosomes (Kumar et al., 2012). SNPs linked to major agro-morphological traits (Deng, 2013; Soto-Cerda et al., 2013, 2014; Xie et al., 2018; Saroha et al., 2022b), oil quality attributes (Soto-Cerda et al., 2014, 2018; You et al., 2018b), fiber length and plant height (Xie et al., 2018), mucilage and hull content (Soto-Cerda et al., 2018), and disease resistance (He et al., 2019a,b) have been identified in flax through GWAS. For improving abiotic stress tolerance, GWAS has been reported in many recent studies to identify potential SNPs for different traits such as oil content, yield, or improved stress tolerance indices in sunflower (Mangin et al., 2017), maize (Millet et al., 2016; Shikha et al., 2017), sorghum (Lasky et al., 2015; Badigannavar et al., 2018; Spindel et al., 2018), rice (Guo et al., 2018), and sesamum (Dossa et al., 2019). Although, a number of genes were discovered and functionally characterized for their role in abiotic stress tolerance in flax, for instance, NAC-domain transcription factor genes (*LuNAC*s) associated with drought, salinity, cold and heat (Saha et al., 2021), putative heat shock factor (*HSF*) candidate genes for high temperature tolerance (Saha et al., 2019), transporter gene family detoxification efflux carriers (*DTX*)/multidrug and toxic compound extrusion (*MATE*) to mediate the response to abiotic stresses (Ali et al., 2020), and aquaporin (*AQP*) gene family in improving drought tolerance (Shivaraj et al., 2017), however, the progress is relatively slow in flax compared to other crops. Regarding biotic stress, Asgarinia et al. (2013) conducted QTL-analysis for powdery mildew resistance and detected loci by homology search in the whole-genome sequencing database using information about nucleotide sequences of ESTs and ВАС-clones. The *de novo* genome of flax rust pathogen *Melampsora lini* was sequenced and assembled and 16,271 putative protein coding genes were identified (Nemri et al., 2014). This could help to understand the previously unknown facts about number of virulence effectors, their function and degree of conservation. He et al. (2019a) conducted GWAS to identify genetic regions associated with pasmo resistance in 370 flax accessions of Canadian core collection and detected 258,873 SNPs using GBS. They identified 500 putative QTL, 45 of which spanned 85 resistance genes. Further, based on orthology with genes of *Arabidopsis thaliana*, two candidate genes, *Lus10031043* and *Lus10020016* for flax resistance to this pathogen were detected. Recently, You et al. (2022) performed both GWAS and GS analyses in 447 flax accessions comprising 372 core collection accessions and 75 breeding lines which were evaluated for powdery mildew resistance for 5–8 years across three locations. They identified a total of 349 QTNs (of which 44 were highly stable large-effect QTNs) and 445 candidate resistant gene analogs (RGAs) associated with powdery mildew resistance in flax. Interestingly, 45 of the identified QTNs were in RGAs of which 14 QTNs were with large effect (*R*^2^ = 10%–30%). Table 1 enlists various QTN/QTLs linked to major abiotic and biotic stresses in flax. However, much work has been done on agronomic and quality evaluation work while little attention has been paid to high throughput sequencing and GWAS for response to climatic threats and pathogen attack in flax. Therefore, comprehensive physiological, biochemical and molecular evaluation under different stress regimes followed by structural and functional genomics strategies as outlined in Figure 3 is required for improving biotic and abiotic stress tolerance in flax.

**Table 1:** Quantitative trait nucleotides/loci identified by Genome wide association studies for major abiotic and biotic stresses in flax.

Trait	QTN/QTL	Candidate gene	Function	References
Stress tolerance index	*Lu6-17,376,408*	*Lus10019811* (probable cinnamyl alcohol dehydrogenase 1)	Drought tolerance	Soto-Cerda et al., 2020
		*Lus10019781* (L-ascorbate peroxidase)	Enhanced salt tolerance, drought, and cold tolerance	
	*Lu14-23,517,150*	*Lus10014978* (aquaporin PIP2-2)	Drought tolerance	
Total root length stability	*Lus-20,209,630*	*Lus10039723* (IAA amido synthetase GH3.6)	Response to stress and root development	
		*Lus10039747* (diacylglycerol kinase 5)	Cold and drought stress tolerance	
	*Lu6-19,733,117*	*Lus10021019* (allene oxide synthase 3)	Stomatal closure and drought tolerance	
		*Lus10020997* (S/T protein kinase SRK2E)	Response to water deprivation and regulation of stomatal closure	
Total root volume stability	*Lu6-15,961,789*	*Lus10016017* (catalase isozyme C)	Promotes drought stress tolerance and response to water deprivation	
Root surface area stability	*Lu5-4,774,423*	*Lus10034840* (calcium transporting ATPase 9, plasma membrane type)	Pollen development	
	*Lu6-15,939,492*	*Lus10016017* (catalase isozyme C)	Response to water deprivation, promotes drought stress tolerance and recovery	
Bundle weight under drought stress	*Chr9:4203006*	*Lus10040333* (3-ketoacyl-CoA synthase 19)	Drought tolerance and biomass related traits	Sertse et al., 2021
		*Lus10040335* (ankyrin repeat-containing protein ITN1)	Salt and drought susceptibility index and biomass related traits	
	*Chr8:16534117*	*Lus10004554* (poltergeist like 1)	Root and Shoot development	
	*Chr12:6352775*	*Lus10016846* (two-component response regulator ARR1-related)	Shoot development and drought tolerance	
		*Lus10016831* (early growth response gene 1)	Seed development and drought tolerance	
Canopy temperature under drought stress	*Chr2:23123754*	*Lus10013240* (xyloglucan endotransglucosylase/hydrolase protein 27)	Leaf size, veins, and drought susceptibility index	
	*Chr3:9279281*	*Lus10019365* (stromal cell derived factor 2)	Heat stress and better stress tolerance indices	
	*Chr9:18937269*	*Lus10024816* (cytochrome p450, family 81, subfamily d, polypeptide 8)	Moisture stress tolerance	
Seeds per boll	*Chr9:15446958*	*Lus10021766* mitogen-activated protein kinase kinase kinase 5	Drought susceptibility index	
Grain yield	*Chr11:3972867*	*Lus10042229* (CBL-interacting protein kinases)	Drought response	
		*Lus10042231* (translocon at the inner envelope membrane of chloroplasts 110)	Heat shock and drought susceptibility index	
Thousand seed weight under drought stress	*Chr1:7029139*	*Lus10029127* (Kelch repeat F-box)	Ovule development and stress tolerance index	
		*Lus10029115* (ribosomal pentatricopeptide repeat protein 4)	Seed development and stress tolerance	
	*Chr12:10910146*	*Lus10030137* (nuclear factor Y subunit A1)	Seed development and drought stress tolerance	
		*Lus10030142* (nuclear pore anchor, translocated promoter region)	Flowering, auxin signaling	
Plant height under drought stress	*Chr5:1375386*	*Lus10029690/1* (cellulose synthase interactive 3)	Flax fiber and stress tolerance index	
		*Lus10029692* (AFI)	Xylem development and stress tolerance	
	*Chr8:2514743*	*Lus10025166* (PIN-LIKES 3)	Plant height and drought tolerance	
		*Lus10025172* (set domain protein 25)	Flowering time	
	*Chr14:205508*	*Lus10009472* (agamous-like 12)	Drought tolerance, root growth	
		*Lus10009476* (C-terminally encoded peptide receptor 2,)	Biomass and N uptake	
		*Lus10009480* (wax inducer 1)	Cell wall structure	
		*Lus10009481* (agamous-like MADS-box protein AGL11)	Plant height	
Yield	*Chr12:20557728*	*Lus10031398* (inositol Monophosphatase 1)	Drought tolerance	
Pasmo resistance	*QTL45/Lu9-6,270,376*	*Lus10031043* (leucine-rich repeat receptor kinase)	Bacterial pathogen associated molecular pattern (PAMP) receptor	He et al., 2019a
		*Lus10031058* (elongation factor)	Effector triggered immunity	
Fusarium wilt resistance	*afB13*	--	--	You and Cloutier, 2020
Powdery mildew resistance	*QPM-crc-LG1 (Lu2698-Lu2712)*	--	--	Asgarinia et al., 2013; You and Cloutier, 2020
	*QPM-crc-LG7 (Lu2810-Lu2832)*			
	*QPM-crc-LG9 (Lu1125a-Lu932)*			
	*-*	*Pm1*		Rashid and Duguid, 2005
	*Lu4-12,432,479*	*Lus10036891*	RGA (WRKY transcription factor)	You et al., 2022
	*Lu5-1,534,998*	*Lus10004727*	RGA (receptor like kinases: RLK)	
	*Lu5-1,535,619*	*Lus10004726*	RGA [toll/interleukin receptor (TIR)-NBS-LRR: TNL]	
	*Lu5-1,569,098*	*Lus10004719*	RGA [toll/interleukin receptor (TIR)-NBS-LRR: TNL]	
	*Lu5-3,006,723*	*Lus10032303*	RGA (WRKY transcription factor)	
	*Lu5-3,224,350*	*Lus10032351*	RGA (receptor like kinases: RLK)	
	*Lu5-13,271,207*	*Lus10029860*	RGA [toll/interleukin receptor (TIR)-NBS-LRR:TNL]	
	*Lu6-1,883,039*	*Lus10017649*	RGA (receptor like kinases: RLK)	
	*Lu12-16,614,785*	*Lus10027903*	RGA (receptor like protein: RLPs)	
	*Lu13-4,531,367*	*Lus10019708*	RGA [toll/interleukin receptor (TIR)-NBS-LRR (TNL)]	
	*Lu14-1,171,479*	*Lus10028639*	RGA (coiled coil-NBS-LRR: CNL)	
	*Lu14-17,203,266*	*Lus10039211*	RGA [toll/interleukin receptor (TIR)-NBS-LRR (TNL)]	
	*Lu15-50,397*	*Lus10007610*	RGA (receptor like kinases: RLK)	
	*Lu15-3,991,048*	*Lus10012678*	RGA (WRKY transcription factor)	

Genomic selection (GS) is a breeding approach that determines the genetic potential instead of identifying specific QTL and thus it overcomes restrictions involved in marker assisted selection (MAS) for speed breeding. GS has the potential to fix all the genetic variation of complex traits contrary to classical plant breeding approach which is slow in targeting the complex and low heritable quantitative traits. That is why it is emerging as promising technique exploiting molecular genetic markers to develop novel markers-based models for genetic evaluation. It involves the precise phenotyping of a selected varied group of genotypes (training population) in multiple environments and genotyping to develop statistical model (GS model) which is employed for the estimation of genomic estimated breeding values (GEBVs) in the breeding population. GS method has many advantages over conventional as well as marker assisted breeding as it deals with minor effect of QTL (Crossa et al., 2017). As a result, GS has been advocated as the most effective method for predicting genetic values for selection by combining all available molecular markers with phenotypic data (Deshmukh et al., 2014; Chaudhary et al., 2015; Abed et al., 2018). GS studies conducted in flax resulted in increased genetic gain per unit time during the breeding cycle (You et al., 2016). They used three bi-parental populations developed by crossing high-yielding, high and low alpha linolenic acid content flax lines for QTL mapping to optimize GS model. He et al. (2019b) developed a high-throughput prediction model of genetic resistance of flax to *Septoria linicola*, which is one of the most accurate genomic prediction model for disease resistance in plants. The latest prediction model by You et al. (2022) has been constructed using 447 flax accessions as a training population and the powdery mildew ratings over 5 years at three locations. All the 349 QTNs identified through GWAS explained 96% of powdery mildew variation showing high predictive ability and the potential of this model in applied in genomic prediction. With the increased genetic and genomic resources in flax, more extensive GS research is expected in the near future which may contribute in releasing new cultivars tailored to specific needs. Presently the more extensive use of GS remains a challenge owing to higher expenses than MAS. However, the availability of low cost, flexible and high-density marker system, cheaper NGS technologies are expected to make the whole genome re-sequencing feasible and cost effective for the GS in near future (Bhat et al., 2016). The current status of GS studies in crop plants, and perspectives for its successful implementation in the development of climate-resilient crops has been reviewed by Budhlakoti et al. (2022) who emphasized that the studies on genetic architecture under drought and heat stress can significantly accelerate the development of stress-resilient crop varieties through GS.

## Transcriptomics

Transcriptome profiling provides a comprehensive overview of gene expression, regulation and helps in identification of key genes involved in stress tolerance mechanism. Various approaches are used to study transcriptome such as expressed sequence tags (ESTs), spotted micro arrays, sequencing along with suppression subtractive hybridization, Affymetrix GeneChips and RNA-sequencing depending upon the availability of genomic resources generated and plant type. With the rapid advancement in next-generation sequencing technologies, RNA-sequencing has become the most efficient, cost-effective and high-throughput transcriptomic method. So far, ample of transcriptomics studies has been carried out in oilseed crops such as flax (Wu et al., 2018), sesame (Dossa et al., 2019), soybean (Leisner et al., 2017), Jatropha (Cartagena and Marquez, 2021), and sunflower (Moschen et al., 2017) to ascertain the effect of drought and salinity.

The flax genome sequencing and availability of genetic maps (Wang et al., 2012; You et al., 2018a; Cullis, 2019; Akhmetshina et al., 2020) laid the foundation for significant number of transcriptomic studies and identification of genes underlying traits of agronomic and economic importance. High-throughput sequencing had been carried out for studying flax response to drought (Dash et al., 2017), alkalinity and salt (Yu et al., 2014, 2016; Dmitriev et al., 2019), metal stress (Dmitriev et al., 2016a; Zyablitsin et al., 2018), and nutrient stress (Melnikova et al., 2015, 2016). Transcriptome study from a moderately drought tolerant flax cultivar (T-397) of Indian origin was conducted by Dash et al. (2017) and expression profiling helped to identify loci/markers for selection of drought resilient varieties. Using transcriptome analysis data, Shivaraj et al. (2017) demonstrated high expression of integral membrane proteins, mostly aquaporins and low expression of integral nodulin-26-like proteins leading to better understanding of their physiological functioning. Another study reported overexpression of drought responsive element binding protein 2A (*DREB2A*) gene imparting drought tolerance in transgenic line of flax *cv. Blanka* (Tawfik et al., 2016). Similarly, for high temperature stress, few genes have been discovered and functionally characterized in flax. Saha et al. (2019) reported the genome-wide identification of 34 putative *HSF* genes from the flax genome. Heat shock factors and NAC domain transcription factors bestow distinct expression patterns under heat stress. Wu et al. (2019a) identified two salt-tolerant genes homologous with *Arabidopsis* Senescence-Associated Gene 29 (*SAG29*) having putative role in enhancing salt tolerance by increasing root length, improving membrane injury and ion distribution. Transcriptome of response of flax to unfavorable soil pH led to revelation of genes with altered expression profiles (Yu et al., 2014; Dmitriev et al., 2016b, 2020; Wu et al., 2019b). Flax response to non-optimal soil acidity (increased pH) and zinc deficiency revealed genes involved in ion transport, cell wall biogenesis and photosynthesis through transcriptomics (Dmitriev et al., 2019). The induction of several pathogen related dominant genes in high pH tolerant flax cultivars were associated to overcome unfavorable effects of reduced Zn content. Melnikova et al. (2016) identified 96 conservative homologs of microRNA belonging to 21 families, and reported the role of seven microRNAs (miR168, miR169, miR395, miR398, miR399, miR408, and lus-miR-N1) in the regulation of gene expression and metabolism in plants under nutrient stress. Changes in the expressions of miR319, miR390, and miR393 associated with significant increase of gene expression in glutathione-S-transferase and UDP-glycosyl-transferase provided insight into putative role of these genes in providing protection against aluminum stress *via* scavenging of reactive oxygen forms and modification of the cell wall (Dmitriev et al., 2017). Similarly, altered expression profiles of lus-miR-N1 and miR399 under phosphate deficiency (Melnikova et al., 2015) were detected. Yu et al. (2016) reported differentially expressed genes (*DEG*s) and saline-alkaline tolerant miRNAs in flax (Lus-miRNAs) for the first time and selected 17 known lus-miRNA and 36 new lus-miRNA after assessment of the DEG profiles to predict the target genes. It was suggested that the miR398 and miR530, coding for superoxide dismutase and transcription factors of the WRK family could play significant roles in flax stress resistance. Genome-wide annotation of miRNAs and phasiRNAs encoding genes along with sRNA transcriptomics (reproductive stage) showed downregulation of phasiRNAs in flax reproductive organs under heat stress (Pokhrel and Meyers, 2022).

Pathogen attack also triggers alterations in the transcriptional and translational profile of plants leading to activation of a number of genes and metabolic pathways as defense mechanism. Kostyn et al. (2012) evaluated the gene response in early stages of infection by Fusarium and identified 47 genes including genes responsible for phenylpropanoid pathway enzymes and antioxidant biosynthesis in flax. Transcriptome of dominant Canadian *cv*. CDC Bethune, an oil type flax resistant to Fusarium wilt and sensitive variety, Lutea identified 100 genes that were differentially expressed in response to early pathogenesis (Galindo-Gonzalez and Deyholos, 2016). Among these, several key genes that are involved in activation of pathogenesis-related (PR) interactions, secondary metabolism and lignin formation had increased transcript abundance in congruence with other pathogenesis related studies done earlier. Similarly, in another study, transcriptome of four fibrous flax cultivars (two resistant and two susceptible) as well as two resistant BC_2_F_5_ populations with respect to Fusarium wilt, showed predominant overexpression of numerous genes involved in defense response such as PR protein encoding genes, ROS production, and related to cell wall biogenesis (Dmitriev et al., 2017). Recently, Boba et al. (2020) reported that upregulation of the terpenoid pathway leading to increased ABA content upon *Fusarium* infection in flax activates the early plant’s response and PR genes especially chitinase and *β*-1,3-glucanase play an essential role for resistance. Earlier study reported that transgenic flax plants overexpressing the β-1,3-glucanase gene showed lower susceptibility to this pathogen (Wróbel-Kwiatkowska et al., 2004). The transcriptomal response of the resistant flax cultivar was found to be quicker and more effective allowing translation to a higher number of activated and repressed genes in response to infection by *F. oxysporum* lini (Boba et al., 2021). The numbers of the differentially expressed PR genes in resistant variety were higher initially (24 hpi) but similar later (48 hpi) in comparison to susceptible variety further established that the degree of the response plays deciding role in the differential resistance reaction, even though the similar qualitative response. RNA-Seq analysis of *M. lini* transcriptome was performed during early establishment of disease in flax and the expression profiles of *Avr*s and effector genes revealed 58 previously uncharacterized genes encoding secreted proteins (Wu et al., 2019b).

Major transcriptomic studies revealing genes that were upregulated/downregulated in response to different abiotic and biotic stresses in flax are listed in Tables 2 and 3. Flax transcriptome sequences and gene expression information are available in NCBI Sequence Read Archive and NCBI Gene Expression Omnibus databases.^1^ Flax microRNA data are deposited in miRbase database, wherein sequences of 124 microRNA of *L. usitatissimum* are presented along with primary and secondary structures and localization in flax genome.^2^ Importantly, there are more research publications regarding tolerance to abiotic stresses in comparison to resistance to the biotic stress in the area of flax transcriptomics, which may be due to targeted traits under breeding programs for specific regions. Most of the transcriptomic studies were limited to only one or two cultivars, however more number of diverse genotypes should be investigated for the comparative analysis and gene function annotation. Study of microRNAs and their role is in the initial stages in flax and several key questions remain unanswered. Further knowledge in this domain will assist scientists to develop artificial microRNA as effective tools to regulate gene expression.

**Table 2:** Global transcriptomic analysis revealing gene expression profiles in response to major abiotic and biotic stresses in flax.

Trait/tissue	Platform/tool	DEGs/DEUs	Key points	References
Flax seed responses to salt stress	Illumina HiSeq 2000	7,736, 1,566, and 452 in alkaline salt stress, neutral salt stress and alkaline stress, respectively	Wax biosynthesis, pathogen-related proteins, and photosynthesis related genes	Yu et al., 2014
	Illumina high throughput sequencing	33,774 Upregulated-18,040 Downregulated-15,734	Provide high resolution gene expression profile	Wu et al., 2019a
Flax leaf responses of drought sensitive and tolerant varieties	PacBio Iso-Seq RSEM	In *cv. Z141* (drought tolerant) Upregulated-3,245 Downregulated-4,167; In *cv. NY-17* (drought sensitive) Upregulated-2,381 Downregulated-3,515	Proline biosynthesis and DNA repair from ROS damage	Wang et al., 2021
Flax (seed, root, and shoot) under drought stress	CombiMatrix 90 K Array	183 Upregulated-72 Downregulated-111	Maintain growth and homeostasis	Dash et al., 2014
Flax seeds under normal and PEG induced osmotic stress	Illumina platform	3,922 Upregulated-1,487 Downregulated-2,432	Biochemical and signal transduction pathway	Wu et al., 2018
Flax seedlings (root) under high soil acidity and aluminum stress	Illumina platform		Compartmentalization of Ca^2+^ in vacuoles and intracellular regulation	Zyablitsin et al., 2018
Flax rust (leaf tissue) under pathogenic stress (*Melampsora lini*)	Illumina genome analyzer II	16,271	Hydrolysis and uptake of nutrients and plant pathogenicity related gene encoding	Nemri et al., 2014
	Illumina HiSeq 2,500	58	Avirulence and effector genes and genes encoding secreted proteins	Wu et al., 2019b
Response to *Fusarium* wilt (*Fusarium oxysporum)*	Illumina HiSeq 2000	100	Reception and transduction of pathogen signals	Galindo-Gonzalez and Deyholos, 2016
		47	Defense response, defense signaling, stress response, and primary and secondary metabolism regulation	Kostyn et al., 2012
	NextSeq 500 high-throughput sequencer (Illumina)		pathogenesis-related protein encoding, ROS production, and cell wall biogenesis	Dmitriev et al., 2017

DEGs, differentially expressed genes; DEUs, differentially expressed unigenes; qRT-PCR, quantitative real time-polymerase chain reaction; RNA, Ribonucleic acid; PEG, polyethylene glycol; ROS, reactive oxygen species; and RSEM, RNA-seq by expectation–maximization.

**Table 3:** Important genes which are upregulated and downregulated in response to various biotic and abiotic stresses in flax.

Trait	Upregulated/downregulated genes	References
Drought	NAC domain proteins	Saha et al., 2021
	Ribulose biphosphate carboxylase/oxygenase activase-2, lipid transfer protein, photosystem I reaction center, EF-tu, Cell wall synthesis genes, r2r3-*MYB* transcription factor, *LEA5*, dehydrin, BRU1 precursor, cell modulin binding heat-shock protein, cytochrome P450 family proteins, histone h2b, *AP2/ERF* domain containing transcription factor, and brassinosteroid insensitive I-associated receptor kinase 1.	Dash et al., 2014
PEG induced osmotic stress	Transcription factors such as *NAC*, *LEA*, *WRKY*, *ERF*, and bZIP	Wu et al., 2018
Salinity-alkalinity	NAC family members, *HSP70*, *WRKY*, *MAPKKK*, ABA, and *PrxR*	Yu et al., 2014
	miRNA targeted genes *Lus-miRNAs*	Neutelings et al., 2012; Melnikova et al., 2014; Yu et al., 2016
	Myb domain protein, Transcription regulators, Auxin signaling F-box, *UBE2* gene, and mitochondrial transcription termination factor family protein	Barvkar et al., 2013; Yu et al., 2016
Heat	Heat shock factors	Saha et al., 2019
	miRNAs and phasiRNAs	Pokhrel and Meyers, 2022
	Heat shock factors (*HSP101B:GUS*)	Young, 2003
	GUS activity showed in sepals, petals, and pistils	Cross, 2002
Nutrient stress	*WRKY, JAZ*, *HARBI1*, and *ING1* families	Dmitriev et al., 2016b
	*lus-miR-N1*, *miR399*, *miR168*, *miR169*, *miR395*, *miR398*, *miR399*, *miR408*, and *lus-miR-N1*	Melnikova et al., 2015, 2016
Aluminum stress	*miR319*, *miR390*, *miR393*, glutathione-S-transferase, and UDP-glycosyl-transferase	Dmitriev et al., 2017
High soil acidity and Aluminum stress	CAX3-Ca^2^+/H^+^ antiporter	Zyablitsin et al., 2018
Fusarium wilt (*Fusarium oxysporum* f. sp. lini)	*PAL*, *PCBER*, *SRG1*, *UGT73C3*, AAA-ATPase *ASD*, mitochondrial (*AATPA*), glucan endo-1,3-beta-glucosidase, *MYB* transcription factors, *ERD* dehydrins, and Auxin-responsive protein *SAUR*, *WKY3*, *WRKY70*, *WRKY75*, *MYB113*, and *MYB108*	Hano et al., 2008; Galindo-Gonzalez and Deyholos, 2016; Dmitriev et al., 2017
*Fusarium culmorum*	*PAL*, *CCR*, *CAD*, *UGT*, and *TD*	Kostyn et al., 2012
Rust (*Melamspora lini*)	*Avrs* and *CWDEs*	Wu et al., 2019b

NAC, nascent polypeptide-associated complex; MYB, myeloblastosis; LEA, late embryogenesis–abundant; ERF, ethylene responsive factors; bZIP, basic-leucine zipper; BRU1, brassino-steroid regulated protein; HSP, heat shock proteins; MAPKKK, mitogen activated protein kinase; AP2/ERF, APETALA2/ethylene responsive factor; UBE2, ubiquitin-conjugating enzyme E2; JAZ, jasmonate ZIM-domain; HARBI1, harbinger transposase-derived nuclease; ING1, inhibitor of growth 1; UGT73C3, UDP-glycosyltransferase 73C3; EF-tu, Elongation factor thermal unstable; GUS, b-glucuronidase; SRG1, senescence related gene 1; PCBER, phenylcoumaran benzylic ether reductase; PAL, phenylalanine ammonia lyase; CCR, cinnamoyl CoA reductase; CAD, cinnamyl alcohol dehydrogenase; UGT, UDP-glycosyltransferase; TD, tyrosine decarboxylase; AVRs, avirulence genes; and CWDEs, cell wall degrading enzymes.

## Metabolomics

Metabolic profiling gives the precise depiction of biological and physiological state of an organism as metabolites are the end products of gene expression and integration of metabolomics has pivotal role in understanding plant systems biology (Weckwerth, 2003; Ghatak et al., 2018; Pontarin et al., 2020). However, the actual size of the plant metabolome being unknown, and owing to the greater diversity of metabolites in plants than other organisms, metabolomic analysis faces some challenges as reviewed by Hall (2006), Schauer and Fernie (2006), and Harrigan et al. (2007). Several analytical platforms have been used to identify and quantify the wide range of primary and secondary metabolites in response to stress, these include a group of well-established analytical techniques, namely, nuclear magnetic resonance (NMR) and mass spectrometry (MS)-based techniques such as GC–MS (Gas Chromatography–Mass Spectrometry), CE-MS (Capillary electrophoresis-Mass spectrometry), LC–MS (Liquid Chromatography – Mass Spectrometry), and FTIR (Fourier transform infrared; Schripsema, 2010; Kaspar et al., 2011; Putri et al., 2013; Simó et al., 2014). NMR requires limited sample preparation and medium to high abundance metabolites are usually detected using this technique. Further, the recent advancements in field strength in NMR superconducting magnets have resulted in improved spectral resolution and detection sensitivity. Contrary to NMR, high and ultra-high resolution mass spectrometers used in current MS-based approaches yield higher sensitivity when analyzing complex plant metabolite mixtures. Ibáñez et al. (2013) presented an overview of recent novel direct ionization or desorption/ionization techniques developed and combined for applications in food metabolomics in their review article. The beneficial effect of metabolites such as lignans, polyunsaturated fatty acids (PUFA), specifically *ω*-3 fatty acids have been well documented for nutritional enhancement and prevention of certain ailments (McCann et al., 2005; Kouba and Mourot, 2011). Flaxseed being an important source of bioactive compounds of interest in human health (lignans and ω-3 fatty acids) and have multitudinous applications in food industry, Ramsay et al. (2014) developed an NMR metabolomics-based tool for selection of flaxseed varieties with better nutrient profile. In addition to metabolomics of nutritional compounds, the detection of the accumulation of many secondary metabolites such as proline, glycine betaine, sugars, and inorganic ions has been reported in oilseeds to help adaption of plant to abiotic stress (El Sabagh et al., 2019). Metabolites, such as *β*-Aminobutyric acid (BABA), have proven role in inducing drought tolerance in *Arabidopsis* (Jakab et al., 2005), spring wheat (Du et al., 2012), apple (Macarisin et al., 2009), rice (Garg et al., 2002), tomato (Cortina and Culianez-Macia, 2005), and potato (Bengtsson et al., 2014). The overexpression of *BABA* resulted in enhanced accumulation of osmoprotectants namely anthocyanins and proline, overexpression of the pathogenesis related genes *PR1*, *PR2*, and *PR5* in *Arabidopsis* (Jakab et al., 2001; Singh et al., 2010; Wu et al., 2010), trehalose biosynthesis induced drought tolerance in tobacco (Romero et al., 1997). In flax, BABA causes accumulation of proline and non-structural carbohydrates and reduction in aspartate content and inorganic solutes in response to water stress (Quéro et al., 2015). Proline and glycine-betaine contents were found to be relatively high under salinity stress in flax (Qayyum et al., 2019) and rice (Cha-um et al., 2006). Total soluble sugars, total protein content and compatible solutes, such as proline, betaine were found to increase with increasing salinity in flax genotypes, suggesting that they may play a role in adjusting osmotic stress under PEG induced water stress and saline-alkaline environments (Guo et al., 2012, 2014; Naz et al., 2016). Differential level of lipid peroxidation and metabolic profile of MDA in wild-type and PLR-RNAi transgenic flax has been reported under salinity and or osmotic stress (Qayyum et al., 2019; Hamade et al., 2021).

Pathogen attack also triggers alterations in the translational profile of plant resulting in synthesis of many secondary metabolites such as flavonoids, catecholamines, polyamines, lignins, terpenoids, tannins, phenolic, and phenylpropanoic acids as defense mechanism. Metabolomics studies have been carried out extensively in rice to find key metabolic products and pathways in response to various biotic stress (Vo et al., 2021). These studies were aimed to understand the induction of defense mechanism involving Pathogen associated molecular pattern (PAMP)-triggered immunity (PTI) and effector triggered immunity (ETI) in model crop rice. The first report to describe metabolites of early flax to Fusarium infection was by Kostyn et al. (2012) who determined the level of metabolites produced in phenylpropanoid pathway (flavonoids and phenolic acids) by GC–MS. Wojtasik et al. (2015) identified for the first-time genes involved in polyamine synthesis pathway and reported increase in content of polyamines putrescine, spermidine, and spermine during *Fusarium* infection in flax. The main polyamine identified was putrescine. Furthermore, differential content of polyamine was measured in response to infection by pathogenic and non-pathogenic *Fusarium* strains in flax which indicate different defense mechanisms. Thus, stress induces drastic changes in the metabolic profile of a plant and therefore complete metabolite profiling may provide valuable insights into stress tolerance mechanisms (Supplementary Figure 1). The prior knowledge of metabolomics in conjugation with other allied omics technologies such as genomics, transcriptomics and proteomics is essential to understand the complete overview of biochemical and molecular mechanisms in response to various biotic and abiotic stress elicitors. However, this is a new research area and no metabolomic databases with reference to environmental stress are available until now.

## Proteomics

Proteomics is the study of the structural and functional characteristics of all proteins in a living organism in real-time. It includes two-dimensional (2-D) gel electrophoresis, mass spectrometry (MS), ELISA, Western Blotting, and matrix-assisted laser desorption ionization-time of flight (MALDI TOF) along with various bioinformatic tools (Baggerman et al., 2005; Gevaert and Vandekerckhove, 2011; Chaudhary et al., 2019). Recent achievement in proteomics has reduced the errors in protein assessment and provided new possibilities for high-throughput proteome analyses. Mostly proteomic investigations have been focused on rice, wheat, barley, maize, potato, and soybean, all of which have whole genome sequences available in public domain. In oilseeds, proteomic studies on Indian mustard (Alvarez et al., 2009), flax (Hradilová et al., 2010; Klubicová et al., 2011), and sunflower (Balbuena et al., 2011) have been reported recently. The proteome analyses revealed that continuous higher level of stress responsive proteins (that includes transcriptional regulators such as SWIB/MDM2 protein, Myb protein, B-Peru-like protein involved in anthocyanin biosynthesis) in tolerant plants help them to cope up with adverse effects of stress compared to sensitive counterpart (Pang et al., 2010; Wendelboe-Nelson and Morris, 2012). Enhanced level of specific proteins, lipoxygenase (LOX), several chaperons (HSP70, HSP90, CPN60-*α*, β, and cyclophilin A), and glutathione-S-transferase (GST) were found in drought tolerant barley and wheat varieties with respect to sensitive counterpart (Kosová et al., 2014). Another study reported reduction in RubisCo (smaller and larger subunits) as well as calcium cycle enzymes such as phosphoribulokinase (PRK), phosphoglycerokinase (PGK) and transketolase in wheat under salt (Caruso et al., 2008), drought (Caruso et al., 2009) and low temperature (Rinalducci et al., 2011). Similarly, changes in OEE1 and OEE2 proteins were frequently found in barley under salt stress (Rasoulnia et al., 2011; Fatehi et al., 2012) and drought stress (Ghabooli et al., 2013). Also, in developing wheat grains subjected to a heat phase, a rise in many minor HSP proteins, as well as HSP82 from the HSP90 family was detected in the endosperm (Skylas et al., 2002; Majoul et al., 2004). Similarly, proteome analysis conjugated with physiological response in two maize varieties resistant to drought stress reported the role of HSP to be important in protecting plants from drought stress (Li et al., 2021b). Lately, Halder et al. (2022) reviewed the role of proteomics for abiotic stress tolerance in wheat and presented a summary of proteomic studies on salinity, drought stress tolerance, and root system architecture conducted in the last decade.

**Figure 4:**
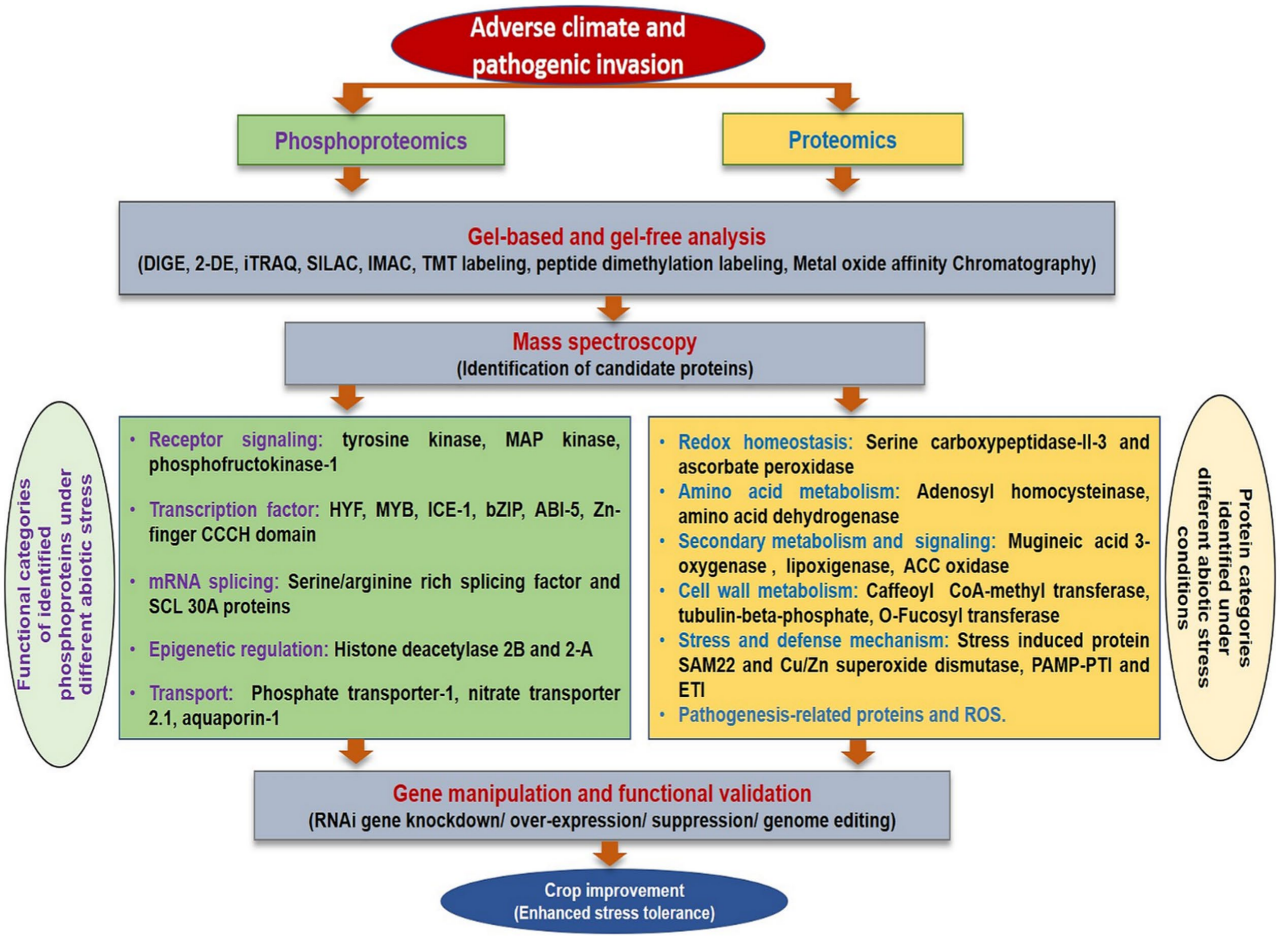
A comprehensive overview of proteomics and phosphoproteomics analysis under different stresses.

Proteomic analysis of biotic stress has been advantageous to describe the proteome of plants and pathogens infected tissues. The global proteomics studies investigating biotic stress responses in rice have been extensively reviewed (Vo et al., 2021) and many potent metabolites responsible for resistance have been enlisted. The changed proteome response in response to biotic stress has been elucidated in many crops such as grapevine resistance to downy mildew (Milli et al., 2012; Palmieri et al., 2012), tomato infected with *Botrytis cinerea* (Shah et al., 2012), avocado resistance to root rot (Acosta-Muniz et al., 2012), and resistance related proteins mainly involved in pathogenesis response were identified. Proteomic analysis has also been used to explore plant-virus interaction to unravel proteins corresponding to enzymes involved in photosynthesis, primary metabolism, and defense (Di Carli et al., 2010). On a similar note, proteomics and phosphoproteomics analyses may assist in identification of candidate protein under various stress conditions in flax (Figure 4). Presently, this domain has been explored to a very limited extent in flax (Hradilová et al., 2010; Klubicová et al., 2011).

## Ionomics

Ionomics is the study of an elemental composition of metal, metalloids and non-metal of the various types of plant species, with a focus on high-throughput detection and measurement (Supplementary Figure 2). Ionomic profile of plant species can be detected using high-throughput technologies such as Inductively Coupled Plasma-Mass Spectrometry (ICP-MS) and Inductively Coupled Plasma-Atomic Emission Spectrometry (ICP-AES; Salt et al., 2008). It provides the important role in understanding the different composition of elements along with their involvement in plant biochemistry, physiology, and nutrition. Plants have evolved with a variety of element uptake abilities owing to numerous soil types and other edaphic factors associated with growth and development (Fujita et al., 2013). Additionally, ionomic profile of a crop is affected by element availability, uptake ability of roots, transport, and environmental stress. A wide range of investigations have been carried out in the realm of ionomics. The ionome of wild and cultivated barley subjected to different salt tolerance levels revealed a substantial negative relationship between the amount of accumulated Na^+^ and metabolites involved in glycolysis and tricarboxylic acid (TCA) cycle (Wu et al., 2013). Studies performed in wheat (Guo et al., 2009) and other grasses, such as *Aneurolepidium chinense* (Shi and Wang, 2005), and *Setaria viridis* (Guo et al., 2011) and flax (Guo et al., 2014) showed that concentration of Na^+^ increases with increasing alkalinity stress as plants accumulate significant levels of Na^+^ in their vacuoles to reduce cell water potential. Under PEG induced water stress in flax, the main inorganic ions involved in osmotic adjustment were K^+^, Na^+^, Ca^2+^, and Cl^−^ thereby increasing drought resistance (Guo et al., 2012). However, no significant differences were observed between the effects of salinity and alkalinity stress on the concentration of Na^+^ and K^+^ in shoots in case of flax (Guo et al., 2014). This suggests that the adaptive mechanism of flax shoots to the alkaline stress may differ from that of other plants such as barley (Guo et al., 2009) and *Chloris virgata* (Yang et al., 2010) where K^+^ concentration of shoots was found to be lower under alkaline stress. Another study in flax showed significant increase in the uptake of Cl^−^, H_2_PO_4_^−^, and SO_4_^2^, whereas the levels of NO^3−^ decreased in flax shoots under salt stress. This depicts that these anions build up in the vacuoles to counteract the input of Na^+^ and together they keep the cell hydrated (Parida and Das, 2005). Also, the concentrations of inorganic anions were much lower under alkali stress than under salt stress with the same osmotic potential, implying that the high pH of alkali stress may block anions such as NO_3_^−^, H_2_PO_4_^−^, and SO_4_^2−^ absorption in flax (Guo et al., 2013, 2014). Application of many inorganic elements can result in enhanced tolerance to abiotic stress, for example, Silicon has proven beneficial against drought, salinity, heat, heavy metals and UV-b (Liang et al., 2007; Pilon-Smits et al., 2009; Deshmukh et al., 2014). Hyperaccumulation of manganese (Mn) in the leaves of grapevine has been reported to delay pathogen spread and thus induction of powdery mildew resistance (Yao et al., 2012). Therefore, studying the elemental profile can aid to better understanding of stress tolerance mechanism. However, ionomics studies are yet to get more attention in flax.

## Phenomics

Phenomics is the study of set of all phenotypes involving genotype, phenotype and environment (GxPxE) interactions in specific environmental conditions using high-throughput analysis (Ichihashi and Sinha, 2014; Tardieu et al., 2017; Zhao et al., 2019; Weckwerth et al., 2020; Ninomiya, 2022). Therefore, phenotype provides ultimate association between environment and plant genotype. In the last decade, advances in sequencing technologies have increased genotyping efficiency, but phenotypic characterization has proceeded more slowly, limiting the identification of quantitative features, particularly those related to stress tolerance (White et al., 2012). Due to complicated biosynthetic processes that address response of plants to external stimuli, phenotyping in response to abiotic stress remains a big challenge (Pratap et al., 2019). In the postgenomic era, the importance of precise phenotyping has become more important owing to dependence of genomic approaches such as GWAS, GS and QTL on the high-throughput phenotyping for the improvement of targeted traits (Walter et al., 2015). Phenomics combined with other omic techniques has the greatest potential for plant breeding. Therefore, non-invasive technologies such as color imaging of biomass, far infrared imaging of the canopy, lidar (includes RBG digital imaging) to assess growth parameters and magnetic resonance imaging (MRI) have been explored to estimate above ground canopy and hidden half (root system) of the plants (Yang et al., 2021), few examples include PHENOPSIS (an automated platform to examine water stress in Arabidopsis (Granier et al., 2006) and soil-filled rhizoboxes for study of root system architecture using RGB imaging in wheat (Bodner et al., 2017), RGB digital imaging for phenotyping of plant shoots (Humplík et al., 2015), infrared thermography to validate role of stomatal conductance in barley and wheat seedlings under salinity stress (Sirault et al., 2009) and chlorophyll fluorescence imaging to screen abiotic stress response in tobacco, canola and cotton (Saranga et al., 2004; Baker, 2008) have been explored. X-ray, computed tomography (CT) and nuclear magnetic resonance (NMR) has been used for 3D visualization of root architecture *in situ*. To automate the analysis of root traits, there has been a proliferation of semi-automated such as SmartRoot, GROWSCREEN_ROOT, EZ-Rhizo, and automated softwares WinRhizo, Root Reader 3D and GiaRoots in recent years. Advanced phenomics platforms for a larger range of crop plants such as state of the art “The Australian Plant Phenomics Facility” (APPF),^3^ multispectral and fluorescence imaging for physiological phenotyping^4^ and many others covering ground-based proximal phenotyping to aerial large-scale remote sensing have been developed. Li et al. (2021a) have elaborated the current developments, configurations, novelties, as well as strengths and weaknesses of diverse high-throughput plant phenotyping platforms in a recent review. Few online databases, such as http://www.plant-image-analysis.org are available to assist users in image processing. Thus, high-resolution IR/NIR cameras, fluorescence imaging systems, laser scanners, hyperspectral imaging systems and high throughput advance plant phenotyping platforms are modern tools to get real time phenome in response to external environment, nutrients and disease. However, deep learning tools are needed to extract phenome information through advanced algorithms from huge datasets generated while phenotyping. In addition, comprehensive management of platforms and softwares are considerable challenges limiting this application to few major crops such as rice, maize and wheat.

## Conclusion

Globally, enormous data are being rapidly generated and annotated to better understand the complicated biological pathways involved in stress tolerance of plants. The availability of diverse genomic resources, such as whole genome sequences, transcriptomes, molecular markers, and linkage maps, has increased significantly in many crops including flax over the last decade. These resources can be efficiently utilized for wider climatic adaptability and biotic stress tolerance in flax through varietal improvement program. Flax being a high value economic crop, finds wide range of uses in the culinary, bioenergy, nutritional, nutraceutical industries. Different omic tools and integrated approaches discussed in the present review provide glimpses of current scenarios and future perspectives for the effective management of abiotic stress and disease resistance in flax. Under integrated approach of omics utilization, the techniques of genomics, transcriptomics, and metabolomics have been employed in flax, but other significant areas such as proteomics, phenomics, and ionomics are yet to be explored. Deeper insight into genetic architecture, signaling pathways, and adaptation under stress through the lenses of different omics technologies are critical to understand the stress response and the underlying regulatory mechanism. Integration of these omics technologies on diverse flax genotypes with substantial trait variation are expected to unravel hitherto unknown factors in flaxseed which would pave way for the breeding of stress tolerant varieties for the larger good.

## Author contributions

BY prepared the initial draft. BY, ON, VK, DW, and SY wrote, edited, and reviewed the original draft. ON and BY helped in the preparation of figures and tables. VK and AK conceptualized the theme. VK, DW, and AK did supervision, reviewing, and editing of the manuscript. All authors contributed to the article and approved the submitted version.

## Funding

This work was supported by funding for the project (No. BT/Ag/Network/Linseed/2019-20) from Department of Biotechnology (DBT), Government of India.

## Acknowledgments

Authors acknowledge the funding support for the project (No. BT/Ag/Network/Linseed/2019-20) from Department of Biotechnology (DBT), Government of India. Authors thank former and present Director, ICAR-National Bureau of Plant Genetic Resources (NBPGR), New Delhi and Heads, DGE, ICAR-NBPGR for facilitation, guidance and inspiration.

## Conflict of interest

The authors declare that the research was conducted in the absence of any commercial or financial relationships that could be construed as a potential conflict of interest.

## Publisher’s note

All claims expressed in this article are solely those of the authors and do not necessarily represent those of their affiliated organizations, or those of the publisher, the editors and the reviewers. Any product that may be evaluated in this article, or claim that may be made by its manufacturer, is not guaranteed or endorsed by the publisher.

## Footnotes

^1^ https://www.ncbi.nlm.nih.gov/geo/

^2^ https://www.mirbase.org/textsearch.shtml?q=Linum

^3^ https://www.plantphenomics.org.au/

^4^ https://www.lemnatec.com/

## Supplementary material

The Supplementary material for this article can be found online at: https://www.frontiersin.org/articles/10.3389/fpls. 2022.931275/full#supplementary-material

Supplementary Figure 1 Schematic diagram showing role of metabolomics in improving abiotic and biotic stress tolerance.

Supplementary Figure 2 Information flow for ionomics and their role in stress response.
