# Supplementary figures and images for: Integrated omics approaches for flax improvement under abiotic and biotic stress: Current status and future prospects
Source: Front Plant Sci. 2022 Jul 25;13:931275. doi: 10.3389/fpls.2022.931275 (PMC9358615; doi:10.3389/fpls.2022.931275)

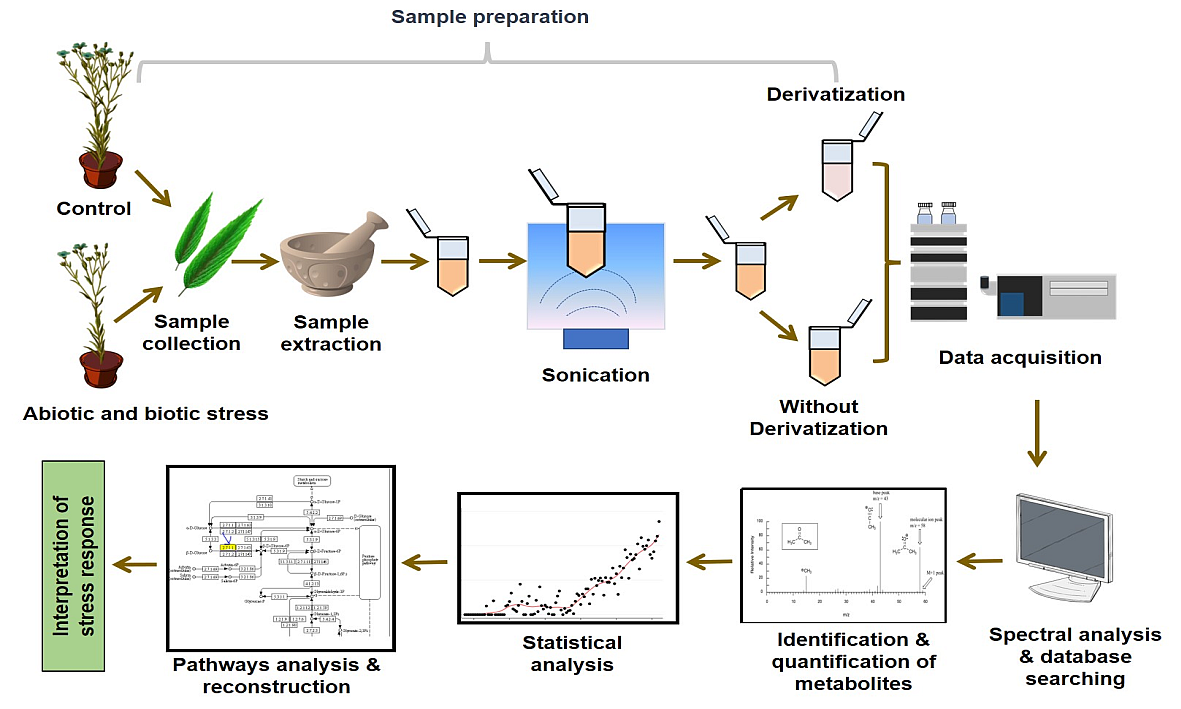

Supplement: Supplementary Figure 1 — Schematic diagram showing role of metabolomics in improving abiotic and biotic stress tolerance. [file Image_1.TIF]

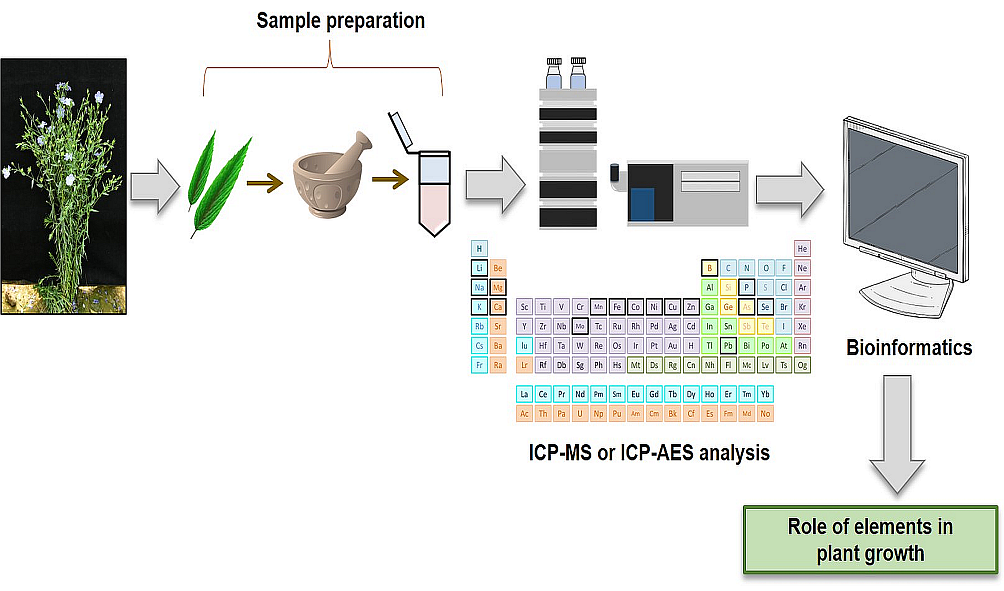

Supplement: Supplementary Figure 2 — Information flow for ionomics and their role in stress response. [file Image_2.TIF]
